# Supplementary material for: Formative evaluation of the acceptance of HIV prevention Artificial Intelligence chatbots by Black gay, bisexual, and other men who have sex with men in the Southern United States: Focus group study
Source: PLOS Digit Health. 2025 Jun 4;4(6):e0000891. doi: 10.1371/journal.pdig.0000891 (PMC12136318; doi:10.1371/journal.pdig.0000891)
Supplement: S1 Text — (DOCX) [file pdig.0000891.s001.docx]

**Interview Guide of Chabot Project - MSM**

**Introduction**

Hello, everyone! Thank you very much for joining our focus group discussion about new strategies for improving HIV prevention in the Southern U.S. We are delighted to have your involvement in this project. In this focus group discussion, we will be looking for your input, thoughts, and opinions regarding an AI chatbot that we are designing to improve access to HIV prevention services, particularly on the awareness and uptake of pre-exposure prophylaxis (PrEP) among black men who have sex with men in the Southern U.S.

The goal of the AI chatbot is to make it easier for men to access PrEP services through your mobile phone. Like how the Uber app made it easier to get a taxi, we want our chatbot to make it easier to get PrEP. We want the chatbot to be as user-friendly, convenient, and acceptable as possible. Your guidance is essential to ensure we develop this effectively. Your feedback will directly shape the chatbot's design, functionality, and user experience.

I’ll be asking some questions and opening the discussion to the group. I am eager to hear from everyone and to learn from you and your experiences. My role is to facilitate the group discussion, ensure equal participation opportunities, and maintain a respectful environment for all participants. There are no correct or incorrect answers. Nor is it required that we achieve consensus. We just want to hear as truthfully as possible from you about the questions asked. In fact, the more opinions the better. We don’t have to agree, but I do ask each of us to be open and respectful of each other.

**Topic 1:** PrEP

**Preamble**: The first topic we’ll be discussing is barriers to PrEP. PrEP is an effective preventive measure for people at high risk for HIV. Yet, PrEP awareness and uptake among Black gay, bisexual, and other men who have sex with men (MSM) in the U.S. South are low. For high risk MSM, new guidelines recommend frequent HIV testing, ranging from every 3 to 6 months, and daily PrEP uptake. HIV testing and PrEP uptake in Black MSM, however, often occurs less frequently.

**Question 1:** What would need to change to improve people’s awareness of PrEP?

Potential prompt:

1. What gets in the way of getting PrEP?

2. What gets in the way of taking PrEP?

**Topic 2:** Chatbot

**Preamble**: Before I ask you the next question, I would like to define a few things to make sure we are all on the same page.

*Have you used a chatbot before? Is there anyone who is not familiar with chatbot?*

**DEMONSTRATE A CHATBOT TO PARTICIPANTS**

using the YouTube video (What are chatbots?)

<https://www.youtube.com/watch?v=pX6zqaEHAdw>

Now, what I would like to do is to pose a question. After you think about it for a few minutes, I will start to make a list of what you think.

**Question 2:** Do you think an AI chatbot can be helpful for promoting PrEP awareness and uptake?

**Question 3:** What functions would you like the AI chatbot to have?

Potential prompts:

- - 1. Ordering HIVST kits, lubes, and condoms.
    2. Giving instruction on the process of getting PrEP.
    3. Finding closest clinics to test for HIV.
    4. Responses in text, pictures, or voice?
    5. Consulting with the doctor using the chatbot?
       1. Access lab results via the chatbot.
       2. Review lab results with the doctor via the chatbot.

**Topic 3:** Digital platform

**Preamble**: You have made a list of the functions that you think are helpful and want to see in the chatbot (GO OVER THE TOP THREE RANKINGS). Next, we would like to discuss which digital platform (e.g., social-networking apps or websites) you want to use to embed the chatbot for sexual health.

Now, like what we did earlier, what I would like to do is to pose a question and after you think about it for a few minutes, I will start to make a list of what you think.

**Question 4:** Where would you expect to find an AI chatbot designed to promote PrEP awareness

and uptake?

Potential prompts:

1. Which type of chatbot participants prefer? A chatbot within a website page or a chatbot within an app?
