# Supplementary material for: Formative evaluation of the acceptance of HIV prevention Artificial Intelligence chatbots by Black gay, bisexual, and other men who have sex with men in the Southern United States: Focus group study
Source: PLOS Digit Health. 2025 Jun 4;4(6):e0000891. doi: 10.1371/journal.pdig.0000891 (PMC12136318; doi:10.1371/journal.pdig.0000891)
Supplement: Attachment — Submitted filename: Response to Reviewers.docx [file pdig.0000891.s005.docx]

November 13^th^, 2024

Editor-in-Chief

PLOS Digital Health

**Object: Resubmission of manuscript for peer review**

Dear Prof./Dr. Leo Anthony Celi,

We hereby submit to the journal PLOS Digital Health for a second round of review, the revised version of this manuscript entitled “**Formative Evaluation of the Acceptance of HIV Prevention Artificial Intelligence Chatbots by Black Gay, Bisexual, and Other Men Who Have Sex with Men in the Southern United States: Focus Group Study**”. In addition to the above revision, we also provide point-by-point responses to reviewers’ comments shown on the table below.

While we await a response from you, we remain yours sincerely.

On behalf of the authors

Dr Zhao Ni, PhD, BMed, RN, FAAN, FAHA

Yale University School of Nursing

Email: [zhao.ni@yale.edu](mailto:zhao.ni@yale.edu)

| Reviewer comment | Response |
| --- | --- |
| REVIEWER I: GENERAL COMMENTS | |
| My comments to the authors are given below. Personally, I feel that the application of artificial functions in the prevention and control of infectious diseases may be a trend. Therefore, I think the selection of this research has certain application value. | Thank you for your comments which have now been addressed as detailed by the below responses. |
| REVIEWER I: MAJOR COMMENTS | |
| 1. The research design is feasible in general. However, the number of cases is small, and the statistical method is simple; In this case, it is recommended to increase the sample size if possible. | Thank you for this comment. We planned to conduct FGDs until reaching data saturation. We concluded the FGDs after 5 sessions when we determined we had reached saturation. After each FGD session, we held debriefings to discuss emerging themes and determine whether additional FDGs were needed. |
| 2. The core concepts and methods in this article need to be explained or described in detail for the reader to understand. | Some more details have now been provided with regards to participant recruitment process as well as on the UTAUT concept used for the study analysis on pages 7, 8 and 9. |
| REVIWER II: GENERAL COMMENTS | |
| See comments/suggestions | Thank you for your comments which have now been addressed below. |
| REVIEWER II: MAJOR COMMENTS |  |
| 1. Add citations to this sentence: "Additionally, AI-driven chatbots have the potential to provide culturally competent and linguistically appropriate..." | A citation (#18) has now been added to the sentence on page 6. |
| 2. Need to standardize how you refer to this: AI chatbots or AI-driven chatbots | Thank you for this comment. We have removed the term “AI-driven” and now use “AI chatbots” consistently throughout the manuscript. |
| 3. Specify how many participants per focus group (range). Also, we all participants living in Southern US. Please clarify. | Three of the groups had 5 participants each while the other two groups had 3 participants each. Yes, all participants were residing in the Southern U.S. |
| 4. In p6, FGD has been spelled out in the study design and participants section but was not initially assigned the acronym. | We have now assigned the acronym. |
| 5. Attach the interview guide as an appendix and make a citation where guide was mentioned. | As per this request, we have now attached the interview guide. |
| 6. Did you provide a brief overview of what AI-chatbots are as part of the FGD? | Yes, we introduced AI chatbots to participants and reinforced the concept through a vide demonstration. This information can be reviewed in the Interview Guide. |
| 7. Specify tools mentioned in the brief survey. Like what depression screening tool was used. | We used the PHQ-2 for depressive symptoms. The rest of the questions were Yes/No or nominal categorical questions that sought the occurrence, type, or frequency of aspects like alcohol, substance use, condomless sex with another male etc. We have now added related information in the Methods section. |
| 8. I do understand that UTAUT was used as an analysis framework to scope the analysis. But I want to check if there were themes that emerged from the data. I think it is important that qualitative studies do not limit themselves to a-priori themes to prevent limitations in interpreting participants' worldviews, especially if you want to compare data between US and Malaysia participants. | The UTAUT was the conceptual framework we used to analyze the study data. This framework, widely used in the field of digital health, constitutes four constructs to evaluate user acceptance (perceived usefulness, perceived ease of use, social influence and facilitating conditions). Using the above constructs only helped in regrouping the themes that were identified (during the first round of thematic coding) but didn’t limit to a-priori themes. That is why despite having some similar themes with the Malaysia study, there were still some other themes that were absent or different. Examples of such themes are: to integrate audiovisual component (vs sending automated reminders) as theme to lower effort expectancy or to improve chatbot’s ability to understand diverse phrasing with culturally sensitive algorithms (not mentioned in Malaysia study). |
| 9. Provide more details on how you go about your qualitative analysis. Like data saturation and steps taken to achieve qualitative rigor (see details here: Shenton, A. K. (2004). Strategies for ensuring trustworthiness in qualitative research projects. Education for information, 22(2), 63-75.) | To ensure credibility of the information acquired during these FGDs, we fostered honesty in participant response by insisting on the respect of the opinion of one another while underlining that there were no wrong or right answers, just diverse opinions all of which were welcomed. Also, we made use of prompts to initiate conversation thus acquiring detailed information on each talking point. Debriefing sessions were held after each FGD by facilitators (research assistant and study PI; ZN) to discussed points raised and to decide on data saturation (the need for a subsequent FGD session). Lastly, themes emerging from analysis were presented to LEN and GAF for a consensus before organizing them into the four constructs of UTAUT. We have gone through the methods section of the paper to ensure that these elements are clearly presented.  We have also endeavored to provide a step-by-step description of study processes to ensure reproducibility of the work. Despite acknowledging the limitation of the study in generalizability, the study findings are very useful in the Southern U.S. and other similar contexts as it provides valuable insights on the challenges faced by Black MSM to PrEP uptake and how AI chatbots can be leveraged to promote PrEP awareness and uptake. |
| 10. Qualitative scholars will feel bad to see thematic analysis as part of "statistical analyses." Statistical analyses is only applicable for quantitative data from the surveys but not the transcripts. | We have now changed “Statistical Analyses” to just “Analyses” to incorporate both statistical and thematic analyses. |
| 11. Easy to say that discrepancies were resolved but please provide proof of interrater reliability (Cohen's Kappa or krippendorff's Alpha) for at least one round of the review. | Despite the benefits of interrater reliability in ensuring consistency, enhancing credibility, and supporting reproducibility, we esteemed that it was not necessary for this study given we proceeded with an exploratory thematic analysis which seek to identify emerging themes in line with challenges of PrEP uptake and how best AI chatbots can be leveraged to improve that. Discrepancy resolution here was thus in line with complementarity of emerging themes (ie themes missed by one coder would be picked by the second coder) and by so doing ensure richness of the data. Furthermore, it should be noted that only JJNN was involved in consolidating and organizing the identified themes in accordance with the four UTAUT constructs. |
| 12. Table 1 style is difficult to read since it is formatted into two columns. Much better to just do the traditional one-column table. | We understand your choice for traditional one-column table presentation here. However, the decision to present this table as such (two-column table) is to avoid presenting a very cumbersome table just for participant characteristics. This table style is thus an alternative to solve that problem. To ease readability, we have put variable names in bold. |
| 13. Can you at least let readers know the age and focus group number of the participants whose quote was used in the results. | The FGDs were conducted in anonymity (participants used pseudonyms not linkable to their survey data). This aligns with our wish to avoid linking talking points to participants since the essence of this study was to identify overall opinions on the matter of AI chatbots for HIV prevention from Black MSM. |
| 14. To balance qualitative and quantitative perspectives, I find it helpful for authors to add numbers when using words that describe quantity, like most, some, few, etc. For instance "Most (70%; n = 15) believed that..." | When we use the term “most” or “unanimously” in the description of our study findings, it doesn’t mean we quantified the talking points that were raised. Instead, it means a point was raised which was backed by other members of the FGD session (a consensus was reached in the session) or was similarly raised in another FGD session. By quantifying these responses, we will be trying to prioritize these talking points, whereas each point made by one person or more is, in our opinion, equally important. |
| 15. This kind of sentences should be placed in the discussion since it is unclear if these were the results of analysis or a discussion of the findings: "Historical injustices and contemporary inequities in healthcare contribute to this mistrust..." | Thank you for this comment. To avoid confusion, we have now deleted the sentence from the text – “Historical injustices and contemporary inequities in healthcare contribute to this mistrust...” |
| 16. The placement of Figure 1 is so far from the point where it was mentioned. | The position of Figure 1 has now been adjusted as requested. |
| 17. What does "Combined with other studies" mean? It does not really inform us how your results is related with previous work. I think it is better to use "Consistent with other studies," (or "In contrast with other studies" if your findings are opposite from previous work). | Thank you for this suggestion. We have now revised the sentence accordingly. |
| 18. Create a subsection heading for study strengths and limitations since the last paragraph of your discussion reflects this. | For the “Discussion” section of the paper, we have now added “Principal Findings” and “Limitations” as subsections. |
| 19. Can you tone down this claim: "The use of AI chatbots will be an acceptable means of communication for behavioral change in relation to HIV prevention in general and PrEP uptake in particular." Perhaps limit this within the context of your study. | We have now limited the conclusion to Black MSM living in the Southern U.S. |
| 20. Add details in funding that the funder had no role the specific aspects of the study. | We have now added the phrase "The funding agency had no role in the design of the study, data collection and analysis, decision to publish, or preparation of the manuscript." |
